# Whole-Genome Sequencing and Mutation Analyses of SARS-CoV-2 Isolates from Indonesia

**DOI:** 10.3390/pathogens13040279

**Published:** 2024-03-25

**Authors:** Sukma Oktavianthi, Aksar Chair Lages, Rinaldy Kusuma, Tri Shinta Kurniasih, Hidayat Trimarsanto, Febi Andriani, David Rustandi, Tandry Meriyanti, Irawan Yusuf, Safarina G. Malik, Juandy Jo, Ivet Suriapranata

**Affiliations:** 1 Mochtar Riady Institute for Nanotechnology, Tangerang 15810, Indonesia; sukma.oktavianthi@mrinstitute.org (S.O.); aksar.lages@mrinstitute.org (A.C.L.); rkusuma@mrinstitute.org (R.K.); tskurniasih@mrinstitute.org (T.S.K.); fandriani@mrinstitute.org (F.A.); irawan.yusuf@mrinstitute.org (I.Y.); safarina.malik@mrinstitute.org (S.G.M.); juandy.jo@uph.edu (J.J.); 2 Eijkman Institute for Molecular Biology, Jakarta 10430, Indonesia; hidayat.trimarsanto@menzies.edu.au; 3 Menzies School of Health Research, Charles Darwin University, Darwin 0811, Australia; 4 Siloam Hospital Lippo Village, Tangerang 15810, Indonesia; david.rustandi@siloamhospitals.com (D.R.); tandry.meriyanti@siloamhospitals.com (T.M.); 5 Department of Biology, Faculty of Science and Technology, Universitas Pelita Harapan, Tangerang 15811, Indonesia

**Keywords:** mutation, genomic surveillance, SARS-CoV-2, whole-genome sequencing

## Abstract

The SARS-CoV-2 infection that caused the COVID-19 pandemic has become a significant public health concern. New variants with distinct mutations have emerged, potentially impacting its infectivity, immune evasion capacity, and vaccine response. A whole-genome sequencing study of 292 SARS-CoV-2 isolates collected from selected regions of Indonesia between January and October 2021 was performed to identify the distribution of SARS-CoV-2 variants and common mutations in Indonesia. During January–April 2021, Indonesian lineages B.1.466.2 and B.1.470 dominated, but from May 2021, Delta’s AY.23 lineage outcompeted them. An analysis of 7515 published sequences from January 2021 to June 2022 revealed a decline in Delta in November 2021, followed by the emergence of Omicron variants in December 2021. We identified C241T (5′UTR), P314L (NSP12b), F106F (NSP3), and D614G (Spike) mutations in all sequences. The other common substitutions included P681R (76.4%) and T478K (60%) in Spike, D377Y in Nucleocapsid (61%), and I82T in Membrane (60%) proteins. Breakthrough infection and prolonged viral shedding cases were associated with Delta variants carrying the Spike T19R, G142D, L452R, T478K, D614G, P681R, D950N, and V1264L mutations. The dynamic of SARS-CoV-2 variants in Indonesia highlights the importance of continuous genomic surveillance in monitoring and identifying potential strains leading to disease outbreaks.

## 1. Introduction

The coronavirus disease 2019 (COVID-19) pandemic caused by a novel β-corona-virus, severe acute respiratory syndrome coronavirus 2 (SARS-CoV-2), has significantly impacted people worldwide. As of September 2023, 770 million confirmed cases and six million deaths were reported [1]. Although many patients were discharged after recovery, there have been cases of reinfection, even among fully vaccinated individuals [2,3]. Furthermore, certain COVID-19 cases are marked by prolonged viral shedding, where the viral RNA remains detected for more than four weeks after initial detection [4].

The genetic makeup of SARS-CoV-2 is a single-stranded positive-sense RNA that spans about 29.9 kb and encodes a polyprotein consisting of four structural proteins, namely, spike (S), envelope (E), membrane (M), nucleocapsid (N), and non-structural proteins (NSPs) [5]. The virus has constantly evolved since its emergence in late December 2019, resulting in multiple variants with varying degrees of infectivity and fatality [6]. Studies have shown that specific mutations, such as those found in the S protein’s receptor-binding domain (RBD), can significantly impact the viral ability to propagate, evade the immune system, and respond to vaccines [7]. Therefore, genomic surveillance is crucial in identifying and monitoring the distribution of these variants and mutations, which can aid in understanding viral real-time evolution and contribute to developing more effective diagnostic tools and vaccines [8].

Previous analyses of SARS-CoV-2 Indonesia isolates have reported the temporal change in predominant lineages during the pre-Delta and Delta outbreaks, raising concerns about the rapid local transmission of SARS-CoV-2 variants [9,10,11,12]. However, more studies are warranted to characterize the mutations within the SARS-CoV-2 genome, which may be involved in infectivity and immune evasion, particularly associated with the Delta variants.

As the world’s fourth most populous country with a vast and dispersed territory, Indonesia faces numerous challenges in improving its genome surveillance capacity [9]. The National Genomic Surveillance Consortium, a joint initiative of the Indonesian Ministry of Health and the Ministry of Research and Technology/National Agency for Research and Innovation, was established in January 2021. This partnership has encouraged extensive technological and public health partnerships, contributing to an increase in the number of SARS-CoV-2 whole-genome sequence uploads to the public database Global Initiative on Sharing All Influenza Data (GISAID) from 1210 in April 2021 to 5791 in August 2021 [13].

As part of Indonesia’s National Genomic Surveillance Consortium, this study investigated the 292 whole-genome sequences of SARS-CoV-2 and associated metadata from COVID-19 patients admitted to routine surveillance at Siloam Hospital Network in eight regions of Indonesia. We aimed to determine the distribution of SARS-CoV-2 clades and mutation patterns in Indonesian isolates. The study period spanned from January to October 2021, covering the Delta variants outbreak and aligning with the national vaccination program that began in mid-January 2021 for the first dose and late July 2021 for the second dose. We further characterized mutation patterns in fully vaccinated patients who contracted the virus. Additionally, we analyzed a larger dataset (*n* = 7515) of published sequences from GISAID between January 2021 and June 2022 to examine the dynamics of SARS-CoV-2 clades distribution over a more extended period. The findings of this study will provide valuable insight into the historical distribution and transmission patterns of SARS-CoV-2 variants in Indonesia, emphasizing the importance of continuous genomic surveillance to monitor viral variants.

## 2. Materials and Methods

### 2.1. Study Design and Population

This retrospective cross-sectional study was conducted to characterize the whole-genomes of 292 SARS-CoV-2 isolates from COVID-19 patients in eight different regions throughout Indonesia, including Bangka-Belitung (*n* = 35), Central Kalimantan (*n* = 52), East Nusa Tenggara (*n* = 23), Greater Jakarta Area (*n* = 58), Jambi (*n* = 38), Maluku (*n* = 36), North Sulawesi (*n* = 32), and West Java (*n* = 18). The samples were collected between 9 January and 22 October 2021, as part of the routine surveillance in the 13 hospitals of Siloam Hospital Network in those regions.

### 2.2. Sample Collection, RNA Extraction, and SARS-CoV-2 Detection

Nasopharyngeal swabs collected by the participating hospital were kept in a viral transport medium and transported to the Mochtar Riady Institute for Nanotechnology (MRIN) laboratory in Tangerang, Banten, at a temperature of 2–8 °C. Upon arrival at the MRIN laboratory, the samples were immediately stored at 4 °C until viral RNA extraction. The RNA extraction was performed using the QIAmp Viral RNA Extraction Kit (Qiagen, Hilden, Germany), following the manufacturer’s instructions. The extracted RNA samples were stored at −80 °C until further use. All samples were tested for the presence of SARS-CoV-2 using the BioA SARS-CoV-2 Real-Time RT-PCR Kit v2.0 (BioAcumen, Singapore, Singapore) in a QIAGEN Rotor-Gene Q Real-Time PCR System (Qiagen, Hilden, Germany). The primers and probes used were designed to detect the ORF1ab and N genes of SARS-CoV-2 RNA, with a specific target sequence of the N gene spanning from 28,936 to 29,033 of the reference sequence (NC_045512.2). Samples with a cycle threshold (Ct) value of <30 were selected for whole-genome sequencing.

### 2.3. Whole-Genome Sequencing

The extracted RNA samples were shipped on dry ice to the National Genome Center, Eijkman Institute for Molecular Biology (EIMB), Jakarta, for whole-genome sequencing. Upon reaching the laboratory, the samples were promptly stored at −80 °C. The sequencing was performed using the Illumina COVIDSeq Test protocol, following the manufacturer’s instructions (Illumina Inc., San Diego, CA, USA) [14]. Briefly, total RNA was subjected to single-strand cDNA synthesis using random hexamers, followed by a multiplex PCR assay using the ARTIC nCoV-2019 v3 primer sets to generate 400 bp amplicons tailing the SARS-CoV-2 genome. Following the Illumina COVIDSeq protocol, the PCR-amplified products were processed for tagmentation and library preparation. All samples were processed in batches on 96-well plates with one no-template control (NTC). These 96 libraries were pooled and quantified using the Qubit dsDNA HS Assay kit on a Qubit 4 fluorometer (Invitrogen, Waltham, MA, USA). The sizes of the fragments were determined using an Agilent 4200 TapeStation System (Agilent, Santa Clara, CA, USA). Sequencing was performed using either the Illumina NextSeq 550 or NovaSeq 6000 systems (Illumina Inc., San Diego, CA, USA).

### 2.4. Data Analysis

The sequencing reads were processed by a custom Ncov19-pipeline [15]. Briefly, raw reads were optically deduplicated using BBMap clumpify.sh script [16], and then were trimmed and filtered using Cutadapt [17], with a Phred score threshold ≥20 and minimum read length ≥ 50 bp. Reads were mapped to the reference sequence Wuhan-Hu-1 (GenBank accession number MN908947) using Minimap2 [18]. SAMTools fixmate and markdup were then used to fix the mate information and remove duplicated reads. Consensus FASTA sequences were assembled using iVar, with consensus bases called the majority base (≥50%) and a minimum depth of 10× at each position [19]. Most of the generated sequences and associated metadata (276/292, 95%) were deposited in the GISAID and accessible at doi.org/10.55876/gis8.230328gx (accessed on 28 March 2023) [20]. Pangolin (version v4.1.3, pangolin-data version v1.16) was employed to assign SARS-CoV-2 Pango lineages [21]. Clade nomenclature was based on GISAID and Nextstrain (Nextclade v2.8.1 tool) [22,23]. Only sequences with an ambiguity score of more than 0.8 were considered for the analysis. NextStrain’s Augur pipeline was employed to construct an unrooted phylogenetic tree [24]. The “treeio” and “ggtree” packages were employed to annotate and visualize the phylogenetic tree. Annotations of mutation were performed using the Coronapp [25]. Furthermore, to provide a comprehensive overview of the SARS-CoV-2 clade distribution in Indonesia, we analyzed a larger Indonesian dataset from the same regions, which included 7515 published sequences on GISAID collected between 1 January 2021, and 30 June 2022. Specifically, these sequences are from Bangka Belitung (*n* = 159), Central Kalimantan (*n* = 224), East Nusa Tenggara (*n* = 280), Greater Jakarta/Jabodetabek (*n* = 4017), Jambi (*n* = 679), Maluku (*n* = 79), North Sulawesi (*n* = 258), and West Java (*n* = 1819). The associated sequences and metadata analyzed are available at doi.org/10.55876/gis8.231201wt (accessed on 1 December 2023) [20]. We performed descriptive statistics to describe the baseline characteristics of the patients and the prevalence of the SARS-CoV-2 variants and mutations. The results were summarized as median (interquartile range, IQR) for continuous variables or number of observations (percentage, %) for categorical variables. The data analyses and graphical presentations were performed using RStudio version 2023.03.1 in R version 4.1.2.

### 2.5. Ethical Issues

This research was carried out as part of routine SARS-CoV-2 testing on patients in participating hospitals. The study followed the guidelines for sequencing the genomes of SARS-CoV-2 Indonesia isolates as part of the National Genomic Surveillance Consortium, established by the Indonesian Ministry of Health and the Ministry of Research and Technology/National Agency for Research and Innovation. This investigation followed strict anonymization procedures and was part of a more extensive epidemiological surveillance effort. Therefore, no additional ethical approval was required for publishing.

## 3. Results

### 3.1. Demographic Data of SARS-CoV-2 Infected Patients

Table 1 describes the baseline characteristics of 292 SARS-CoV-2 patients encompassing different regions of Indonesia. Table S1 shows the characteristics of individuals in each region. The median age of the patients was 32 years old (interquartile range/IQR of 26–45 years old). The majority of the patients were in early adulthood (18–40 years old; 61.3%) and late adulthood (41–65 years old; 29.1%), whereas the proportion of children and adolescents (<18 years old) as well as elderlies (>65 years old) were both 4.8%. The proportion of males and females was 52.1% and 47.9%, respectively. Approximately 19.2% of the patients were hospitalized.

Based on the reported data, we observed a co-occurrence of breakthrough infections (BI), prolonged viral shedding (PVS), and reinfection (RI), as illustrated in Figure S1. A total of 38 patients tested positive for COVID-19 more than 14 days after receiving their second dose of CoronaVac, classified as BI. Of these BI events, 32 cases reported a single event of breakthrough infection (BI), while three had both breakthrough infection and prolonged viral shedding (BI + PVS), and three had breakthrough infection and reinfection (BI + RI). Of the three BI + RI cases, two contracted the first COVID-19 before vaccination, while one tested positive at the time of vaccination. Furthermore, five patients reported PVS, while one reported RI.

The descriptive characteristics of these groups are outlined in Table S2. The median age of patients in the BI, BI + PVS, and BI + RI groups was 26 years old (IQR 24–29 years old), 28 years old (IQR 27–29.5 years old), and 41 years old (IQR 33.5–45 years old), respectively. Additionally, the median age of the PVS patients was 41 years old (IQR 39–45 years old), while an RI patient was 41 years old. Females accounted for 21 (66%) of the BI cases, two (67%) of the BI + PVS cases, and three (100%) of the BI + RI cases. Males are more prevalent (80%) than females (20%) in the PVS group, while the reinfected subject is female.

The median PCR Ct values for the BI, BI + PVS, and BI + RI groups were 18.0 (IQR 16.2–21.3), 16.4 (IQR 15.2–20.1), and 18.1 (IQR 17.7–23.9) for the N gene, respectively. While the median Ct values of the ORF1ab gene were 17.2 (IQR 14.9–20.2), 15.6 (IQR 14.4–18.5), and 17.5 (IQR 17.3–22.7) for BI, BI + PVS, and BI + RI groups, respectively. Median Ct values in PVS were 14.7 (14.4–18.3) for the N gene and 14.7 (13.0–18.2) for the ORF1ab gene. Meanwhile, Ct values in a subject with RI were 15.3 for the N gene and 16.8 for the ORF1ab gene (Table S2). Based on these observations, we noted a discernible difference in the Ct values among these groups. However, we could not conduct a statistical analysis to explore these differences further due to the small sample size.

### 3.2. Phylogenetic Analysis

**Figure 1:**
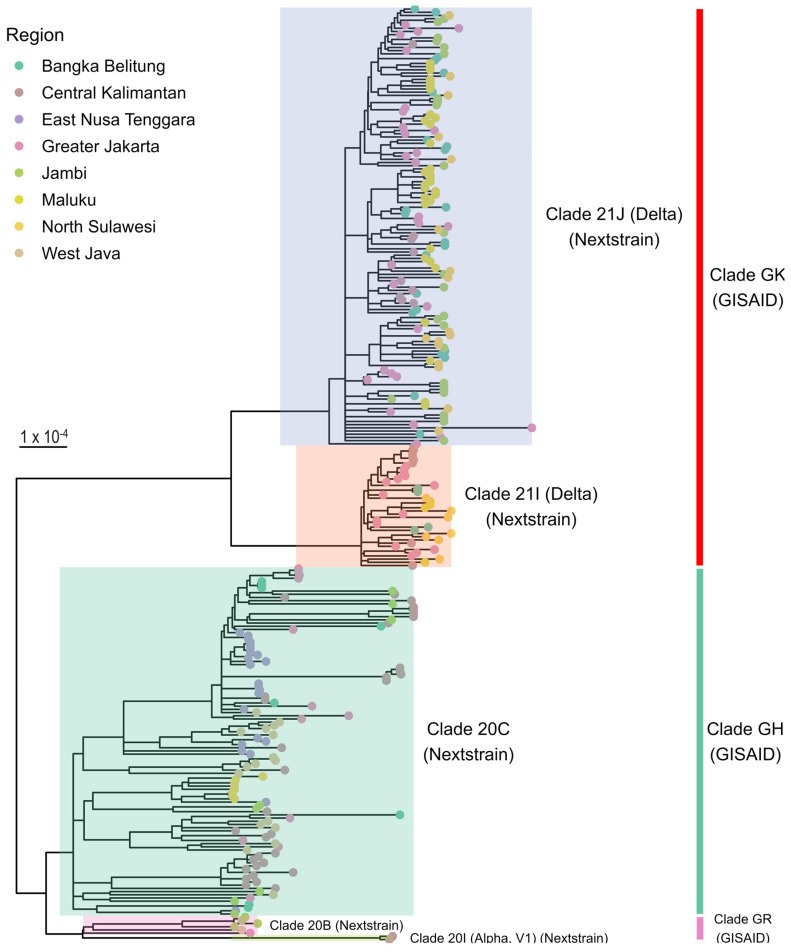
A phylogenetic analysis of 292 whole-genome sequences of SARS-CoV-2 from eight regions across Indonesia (January to October 2021). The unrooted phylogenetic tree was constructed using NextStrain’s Augur pipeline. Tree annotation and visualization were performed using the “treeio” and “ggtree” packages in R. Each tip color represents the sample location. The tree is shaded and labeled to correspond to the Nextstrain and GISAID clades.

The phylogenetic analysis of the 292 SARS-CoV-2 whole-genome sequences shown in Figure 1 indicated that the SARS-CoV-2 genomes from various parts of Indonesia could be classified under three major clades, namely, GK, GH, and GR, as identified by GISAID, or five clades, namely, 21J (Delta), 21I (Delta), 20C, 20B, and 20I (Alpha), as determined by Nextstrain. Of note, GISAID clades GK and GR were divided into Nextstrain clades 21J (Delta) and 21I (Delta), as well as Nextstrain clades 20B and 20I (Alpha), respectively.

### 3.3. Distribution of SARS-CoV-2 Clades and Lineages

**Figure 2:**
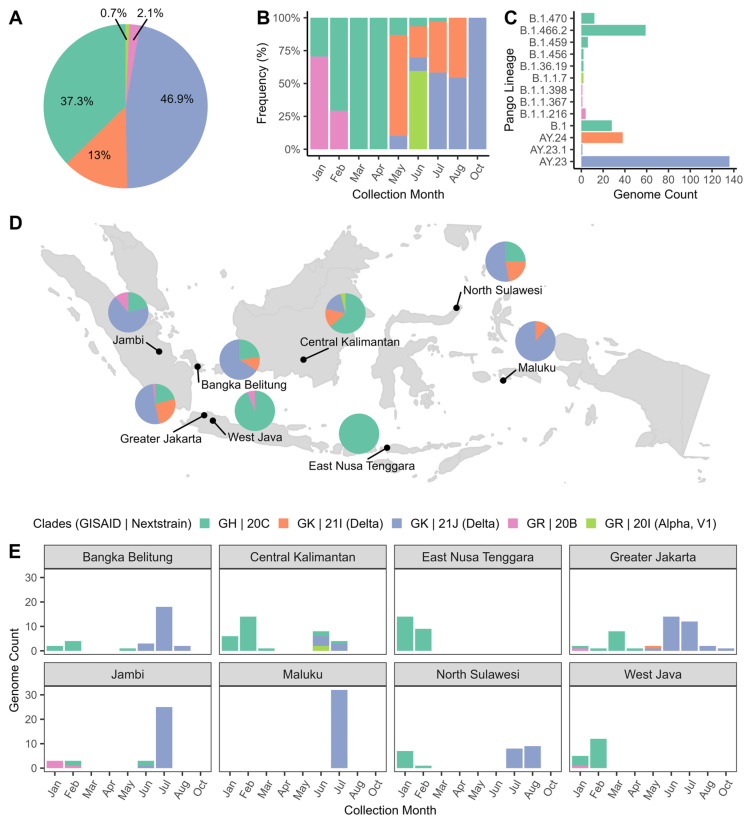
The distribution of SARS-CoV-2 clades and Pango lineages in the 292 Indonesian isolates. The GISAID and Nextstrain clades distributions were shown in (**A**) all samples and (**B**) stratified by collection month. (**C**) The proportion of Pango lineage. (**D**) The distribution of GISAID and Nextstrain clades across Indonesian regions. (**E**) The number of GISAID and Nextstrain clades found in each region of Indonesia.

Figure 2 displays the distribution of SARS-CoV-2 clades and Pango lineages in Indonesia during the study period (January–October 2021). The Delta clade (GK | 21J) was the most widespread in 46.9% of all samples. The GH | 20C clade was a close second at 37.3%, with GK | 21I at 13% and GR | 20B accounting for less than 5% of all samples. Data analysis by specimen collection month indicated an upward trend of clade GH | 20C from January to April 2021, while clade GR | 20B experienced a significant decline. From May to October 2021, clade GK | 21I was the predominant clade. Clades GK were present in all regions except East Nusa Tenggara and West Java. In addition, the top three Pango lineages circulating during the study period were lineage AY.23, B.1.466.2, and AY.24. These findings provided valuable insights into the molecular epidemiology of SARS-CoV-2 in Indonesia during the study period.

We comprehensively analyzed 7515 published sequences on GISAID from the same regions in Indonesia to understand the distribution of SARS-CoV-2 clades over an extended period between 1 January 2021, and 30 June 2022 (Figure S2). In line with our dataset, we observed that the spike in Delta variants began in May 2021 and remained dominant until November 2021. However, Delta’s frequency started to decline in December 2021, replaced by the Omicron variants (GRA | 21K (BA.1) and GRA | 21L (BA.2) clades), which predominated the circulating SARS-CoV-2 variants since December 2021. Furthermore, we identified the emergence of the sub-variant of the Omicron, namely, the GRA | 22B (BA.5) clade was identified in January 2022, the GRA | 22A (BA.4) clade was found in May 2022, and the GRA | 22C (BA.2.12.1), GRA | 22F (XBB), and GRA | 22D (BA.2.75) clades were found in June 2022. These Omicron variants have become the most prevalent SARS-CoV-2 strains circulating in the population.

### 3.4. Mutation Analyses of SARS-CoV-2 Genomes

**Figure 3:**
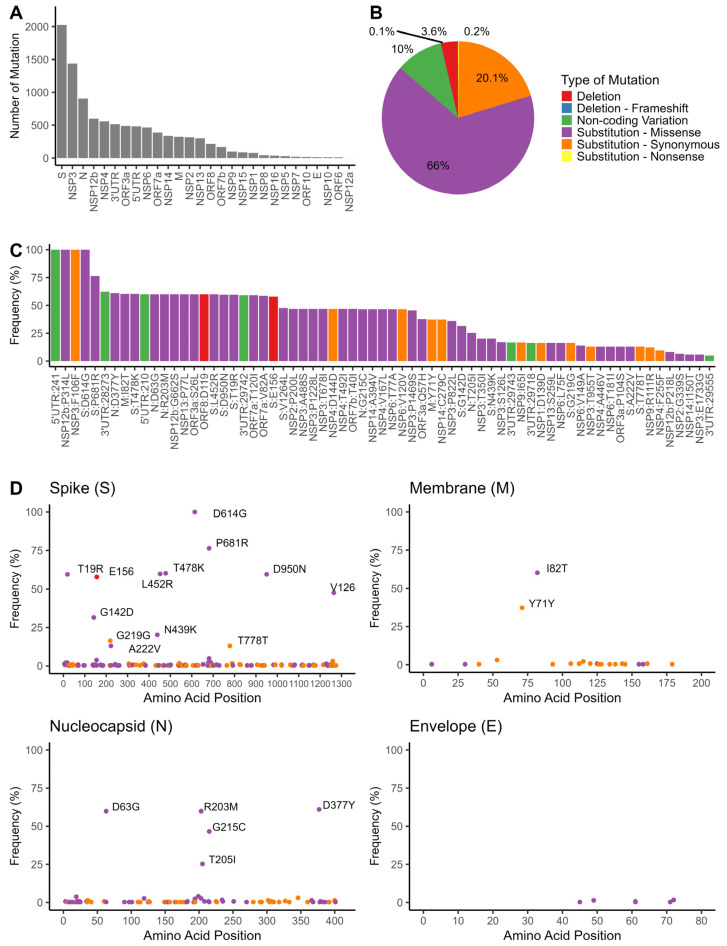
The distribution of mutational events in the 292 SARS-CoV-2 genomes from the Indonesian isolates: (**A**) The number of mutation events across the SARS-CoV-2 non-coding and protein-encoding regions. (**B**) Distribution of type of mutations found in all samples. (**C**) The distribution of most frequent mutation events (frequency > 5%) of SARS-CoV-2 with annotated protein changes. (**D**) The distribution of mutation events in the sequence of SARS-CoV-2 structural proteins, including Spike (S), Membrane (M), Nucleocapsid (N), and Envelope (S). Mutations with a frequency of >5% are indicated. Purple dots represent the missense substitutions, while orange dots depict the synonymous substitutions.

The mutational events were mostly observed in the spike (S) protein region, followed by NSP3 and nucleocapsid (N) protein (Figure 3A). As shown in Figure 3B, missense mutations were the most prevalent type of mutations within the SARS-CoV-2 genome isolated from Indonesia, accounting for 66% of all mutations. In addition, synonymous mutations accounted for 20.1% of total mutations, non-coding variations accounted for 10%, and deletion polymorphisms comprised 3.6% of the observed mutations (Figure 3B). Certain variations, including C241T (5’UTR), P314L (NSP12b), F106F (NSP3), and D614G (S), were shared among all isolates (Figure 3C). Figure 3D depicts the distribution of mutations in the structural proteins of the SARS-CoV-2 genome. The most common mutations detected in the S protein were D614G (100%) and P681R (76.4%). Other mutations (including T478K, L452R, T19R, D950N, E156, V1264L, G142D, and N439K) were observed at moderate-to-low frequencies (20–60%). The most frequent mutations in the nucleocapsid (N) protein were identified as D377Y (61%), D63G (60%), R203M (60%), G215C (47%), and T205I (25%).

The mutations in the spike and nucleocapsid proteins among SARS-CoV-2 clades are displayed in Figures S3 and S4. Spike mutations D614G and P681R were observed in all clades. The N439K mutation, found in a lower frequency (20%), was exclusively observed in the GH | 20C clade (B.1.466.2 lineage). Additionally, sequences belonging to the Delta GK | 21I and GK | 21J clades carried the E156 deletion along with several substitutions, including T19R, G142D, L452R, T478K, and D950N. We further found that A222V was only present in the AY.24 lineage (Delta GK | 21I clade) and appeared less frequently (13%) in this population. Moreover, the V1264L mutation was primarily found in the 21J clade (AY.23 and AY.23.1 lineages) and in two sequences from the B.1.470 lineage collected in February 2021 (Figure S4). Additionally, several mutations in the N protein with varying frequencies (25–60%), such as D63G, R203M, T205I, G215C, and D377Y, were present. Of these mutations, D63G, R203M, and D377Y were found in the Delta GK | 21I and GK | 20J clades, while G125C was only present in the GK | 21J clade. Furthermore, two consecutive mutations, R203K/G204R (RG203KR), were present in the Alpha variant B.1.1.7 lineage (GR | 20I clade), as well as in the B.1.1.216, B.1.1.398, and B.1.1.367 lineages (GR | 20B clade).

### 3.5. Mutation Patterns among Patients with Breakthrough Infection, Reinfection, and Prolonged Viral Shedding

The distribution of clades and lineages for the studied groups is presented in Table S2. Among the 32 sequences in the BI group, 18 (56.3%) belonged to the Delta GK | 21I clade (AY.23 lineage), 12 (37.5%) were in the Delta GK | 21J clade (AY.24 lineage), and 2 (6.2%) were classified under the GH | 20C clade (B.1.466.2 lineage). Additionally, all sequences from the PVS and BI + PVS groups were classified under the Delta GK | 21J clade (AY.23 lineage). Out of the three sequences from the PVS+RI group, two (67%) belonged to the Delta GK | 21J clade (AY.23 lineage), while one (33%) was classified under the Delta GK | 21I clade (AY.24 lineage). Furthermore, one sequence from the RI case was classified under the GH | 20C clade (B.1.466.2 lineage).

**Figure 4:**
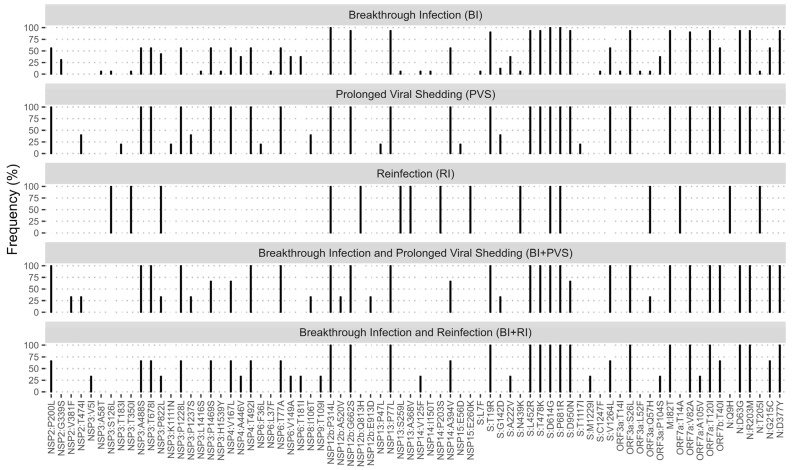
The pattern of amino acid substitutions in patients with breakthrough infection (BI), prolonged viral shedding (PVS), and reinfection (RI). Of the 292 patients, 32 reported BI, five reported PVS, one reported RI, three reported combined BI + PVS, and three reported combined BI + RI. Amino acid substitutions with frequency of >5% were included.

Detailed mutation patterns in BI, PVS, RI, BI + PVS, and BI + RE groups are illustrated in Figure 4. We observed that the BI, PVS, RI, BI + PVS, and BI + RE groups had variations of amino acid substitution patterns, aside from P314L (NSP12b), D614G (S), and P681R (S) that were shared in all groups. We also observed that mutation patterns in BI, PVS, BI + PVS, and BI + RE groups were mainly characterized by Delta signature mutations in the S protein, such as T19R, L425R, T478K, D950N, and V1264L. These groups also shared the I82T mutation in the M protein and the D63G, G215C, R203M, and D377Y mutations in the N protein. BI and BI + RE groups had A222V, which was less frequent in PVS and BI + PVS groups. However, G142D substitution was found in BI, PVS, and BI + PVS groups but not in others. Meanwhile, the RI case showed a distinct mutation pattern in the structural proteins, including S:N439K and N:T205, commonly found in sequences belonging to the GH | 20C clade, along with additional mutation N:Q9H.

## 4. Discussion

The COVID-19 pandemic remains a significant global health issue. Given its mutability, new and more potent variants of SARS-CoV-2 can arise rapidly. Monitoring those variants is, therefore, crucial to understanding their transmission patterns and identifying potential new strains that may cause infection outbreaks [8]. Therefore, we investigated the historical distribution of SARS-CoV-2 variants in selected populations across Indonesia between January and October 2021 by utilizing comprehensive whole-genome data.

The genomic surveillance study revealed the dynamic of SARS-CoV-2 clades distribution over time in Indonesia (Figure 2B). From January to April 2021, indigenous lineages, such as B.1.466.2 and B.1.470, were more prevalent. As of May 2021, however, the Delta variants have become the dominant strain. This trend aligns with previous studies that found Indonesia-associated variants (B.1.466.2 and B.1.470) were widespread before the Delta outbreak. These local variants were subsequently outcompeted by Delta lineages AY.23 and AY.24 [9,10,11,12].

In June 2021, sequences from Central Kalimantan showed the presence of the Alpha variant (B.1.1.7). Although this variant was already widespread worldwide by that time and it was first identified in Indonesia in January of the same year, there have been only a few reports on the Alpha variant in the Indonesian population [10,12,26]. The introduction of the B.1.1.7 variant in Central Kalimantan was likely due to travelers from neighboring Asian countries where this variant was prevalent.

An analysis of 7515 published SARS-CoV-2 sequences collected between January 2021 and June 2022 enabled us to understand the distribution of clades in the general population (Figure S2). Our findings showed that the distribution of clades in the larger population (Figure S2, *n* = 7515) is consistent with our dataset (Figure 2B, *n* = 292), indicating that the Delta variants started to spike in May 2021 and became the predominant strain until November 2021. Nevertheless, with a longer timeframe, we observed the emergence of the Omicron GRA | 21K (BA.1) and GRA | 21L (BA.2) clades in December 2021, along with a decrease in Delta variant frequency. As the months progressed, the Omicron variant continued to spread rapidly and became increasingly dominant, with several subvariants also emerging, including the GRA | 22A (BA.4), GRA | 22C (BA.2.12.1), GRA | 22F (XBB), and GRA | 22D (BA.2.75) clades. The Omicron variants have caused global concern due to their increased transmissibility and high immune evasion ability established by vaccination or previous infection by other variants [27]. These variants carried several mutations in the RBD region and N-terminal domain (NTD) that affected the neutralizing antibodies binding [28,29]. This shift in the virus’s genetic makeup has significant implications for public health, and it is essential to continue monitoring SARS-CoV-2 variants to develop effective strategies to combat the spread of the virus.

This study identified several mutations of concern present in the Spike (S) protein, including L452R, T478K, D614G, and P681R (Figure 3D). Mutations L452R and T478K located in the RBD region have been shown to increase viral transmissibility and reduce the effectiveness of neutralizing antibodies [30,31]. One of the most common mutations, D614G, was associated with efficient human-to-human transmission but not increased disease severity [32,33,34]. Mutation P681R, located at a furin cleavage site that separates the spike 1 (S1) and S2 subunits, was common in the Delta variants. The mutation may enhance viral entry through the cell surface [35]. The other Spike mutations associated with the Delta variants, T19R, G142D, and D950N, were also identified. These mutations have been associated with reduced sensitivity of Delta variants to antibody neutralization [36].

Furthermore, the Delta sequences carried a deletion of E156 that may increase the virus’s infectivity and ability to evade the immune system [37]. Another Delta-specific mutation, A222V, was identified in the AY.24 lineage and was associated with a slight increase in the S protein’s affinity for the ACE2 host receptor [38]. Furthermore, the V1264L mutation, located in the cytoplasmic tail of the S protein, was identified in various clades, including Delta GK | 21J (AY.23 and AY.23.1 lineage) and GH | 20C (B.1.470 lineage) (Figure S3). This mutation served as an intracellular targeting motif that regulates virion assembly of SARS-CoV-2 [39]. While primarily observed in the Delta variant, the occurrence of this mutation in a non-Delta lineage suggested that it may also be present in other genetic backgrounds of SARS-CoV-2.

The N439K Spike mutation was previously identified as a signature motif for the B.1.446.2 lineage originating from Indonesia [9,10]. Our observations also revealed that this mutation is exclusively present in the B.1.466.2 lineage of the GH | 20C clade. This particular mutation is among the most prevalent RBD mutations globally. It has raised concerns due to its potential to enhance infectivity by forming a new salt bridge with human ACE2, and increasing resistance to neutralizing antibodies [40].

The nucleocapsid (N) protein plays a vital role in virus replication and transcription, immune response regulation, and packaging of viral genomes in new virions [41]. Mutations in the N protein can negatively affect antibody binding, worsening the disease’s severity [42]. Our study also identified frequent amino acid changes, including D63G, R203M, T205I, G215C, and D377Y, occurring at frequencies ranging from 25% to 60% (Figure 3D). D63G, R203M, and D377Y were present in all GK | 21I and GK | 20J clades of Delta variants, while G125C was only found in the GK | 21J clade (Figure S4). The mutation of D377Y first appeared between January and March 2021, in which its frequency significantly increased between June and July 2021. Meanwhile, D63G, G215C, and R203M mutations emerged in May 2021 as the Delta outbreak progressed (Figure S5). D63G has been linked to increased viral loads, accelerated viral replication, and a higher risk of ICU admission [43,44]. T205I was only present in GH | 20C clade sequences. Additionally, two consecutive mutations, R203K/G204R (RG203KR), were identified in the Alpha B.1.1.7 lineage (GR | 20I clade), as well as the B.1.1.216, B.1.1.398, and B.1.1.367 lineages (GR | 20B clade) (Figure S4). These mutations increase viral replication, enhancing the Alpha variant’s infectiousness, fitness, and virulence [45].

Our study revealed several mutations in the viral genome’s regulatory regions and non-structural proteins. All isolates carried these three mutations: P314L substitution in the RNA-dependent RNA polymerase (NSP12b) protein, C241T variation at 5’UTR, and a synonymous variation F106F in NSP3 (Figure 3C). The P314L mutation may affect SARS-CoV-2 RNA replication and polymerase activity [46]. The C241T variation, on the other hand, had been linked to decreased viral replication efficiency, higher recovery rates, and reduced mortality rates in SARS-CoV-2 cases worldwide [47]. Although the F106F mutation was considered silent, it was associated with an altered miR-197-5p target sequence, a host-specific miRNA necessary to defend against coronaviruses [48]. Studies have demonstrated a strong correlation between these non-structural protein mutations and the spike mutation, indicating a potential interaction that could impact viral pathogenesis and evolution [49].

During the COVID-19 pandemic, individuals who had been fully vaccinated or had a previous SARS-CoV-2 infection still had a risk of contracting the virus, known as breakthrough infection (BI) and reinfection (RI) [2]. Our study found that most BI cases were linked to Delta lineages in June–August 2021 (Table S2). Furthermore, a few cases (6%) during the pre-Delta outbreak (March 2021) were associated with the indigenous Indonesia B.1.466.2 lineage that was dominant during that period. This finding aligns with previous research suggesting BI cases are not limited to any specific viral variant and can be influenced by various factors [2,3].

This study also revealed that three subjects with a prior history of COVID-19 were reinfected with SARS-CoV-2 after completing the second vaccination dose, classified as the BI + RI group (Figure S1). The virus variants associated with these cases were identified as Delta variants (AY.23 and AY.24) (Table S2). However, since this study did not examine specimens of the first infection, we could not confirm their virus variants. Nonetheless, given that their first infections occurred in March–April 2021 (Figure S1), it is likely that their first infections belonged to the non-Delta lineage. Even after receiving booster vaccination, reinfection with Delta variants further emphasizes Delta variants’ high infectivity and transmissibility.

Previous studies have shown that although the vaccine has high initial efficacy, its effectiveness against the Delta variant decreases over time [50,51], possibly due to emerging mutations in the S protein that enhance the virus’s immune evasion ability [52]. This study revealed that BI, RI, and BI + RI cases were characterized mainly by Delta’s signature mutations, including T19R, L452R, T478K, D614G, P681R, D950N, and V1264L (Figure 4). These mutations have been demonstrated to enhance viral infectivity, fitness, and transmissibility and to reduce the antibody neutralization elicited by infection or vaccination [31,33,53,54,55].

It has been observed that some COVID-19 patients, especially immunocompromised patients and patients with advanced age and comorbidities, experience prolonged viral shedding (PVS), where viral RNA remains detectable for over a month [56,57,58]. This study found that all PVS-related cases, including those with BI, carried the Delta AY.23 lineage (Table S2). Delta signature mutations associated with viral infectivity and immune evasion are present in these cases, including T19R, G142D, L452R, T478K, D614G, P681R, D950N, and V1264L. Notably, the G142D mutation was observed in the BI, PVS, and BI + PVS groups (Figure 4). It has been reported that this mutation is associated with a milder Delta infection in Makassar, Indonesia [11]. This mutation affects the super-site epitope in the NTD of the S protein, which binds to NTD-targeted neutralizing antibodies. This change could contribute to the increased infectivity, immune evasion, and breakthrough infections commonly observed in Delta variants [59].

As previously reported, individuals infected with Delta variants have a higher viral load and take longer to test negative through PCR [60]. Nevertheless, the exact mechanism underlying PVS remains unclear. A previous study suggested that the dynamics of the viral shedding, including infectious virus titer kinetics and the SARS-CoV-2 variant’s incubation times, cannot be predicted solely from mutation patterns [4]. A recent study found that different mutations may emerge in immunosuppressed patients during prolonged viral shedding. These mutations could help the virus replicate more effectively and adapt to the host’s selective pressure, improving its fitness in each individual [61].

It is important to note that this study has some limitations. The study focused on the period of SARS-CoV-2 genomic surveillance before and during the Delta outbreak, from January to October 2021. As a result, the Delta variants were the most prevalent variants of concern observed. However, we have expanded our analysis by incorporating a more extensive dataset of published sequences from Indonesian isolates, which covers the period from January 2021 to June 2022 (Figure S2). This analysis allowed us to study the dynamics of SARS-CoV-2 variants over a more extended period, from the Delta outbreak until the emergence of the Omicron variants in December 2021. It is worth noting that reports on the dynamic distribution of SARS-CoV-2 variants from developing countries are limited. Therefore, these findings provide valuable insights into viral genomic changes between January 2021 and June 2022.

Unfortunately, we could not thoroughly analyze the published dataset (*n* = 7515) due to limited information on vaccination status, history of reinfection, and prolonged viral shedding. This limitation prevented us from studying the mutation patterns of the BI, PVS, and RI groups with a larger sample size. Nevertheless, further studies integrating genetic and phenotypic data (such as clinical characteristics, disease severity, and vaccination history) are necessary. It allows us to attain a more comprehensive understanding of diseases and develop more effective treatments.

Furthermore, the surveillance of SARS-CoV-2 in Indonesia, as in other low- and middle-income countries (LMICs), has encountered numerous challenges. Limited financial resources, infrastructure, and trained personnel led to an uneven distribution of sequencing resources across different regions. It resulted in over-sampling in areas with better surveillance infrastructure and resources, while remote regions with limited access to sequencing facilities were under-sampled [62].

Sampling time and frequency could also introduce biases into the sample collection process. The number of confirmed COVID-19 cases and sequence specimens worldwide has consistently decreased since the public health emergency status was lifted in May 2023 [63]. Notably, the number of submissions of Indonesia-originated sequences to GISAID has significantly reduced, with only seven submissions since July 2023 (https://doi.org/10.55876/gis8.231023fo, accessed on 23 October 2023). However, the emergence of new Omicron sub-variants, specifically BA.4 and BA.5, is still a cause for concern [64]. Therefore, it is crucial to continue monitoring and surveillance of any new mutations that may result in another pandemic.

## 5. Conclusions

Our study found that the clades of SARS-CoV-2 in the Indonesian population underwent dynamic changes from January 2021 to June 2022. We identified genetic mutations frequently present in SARS-CoV-2 clades in Indonesia by examining whole-genome sequences. These historical data are hopefully valuable in comprehending the spread of SARS-CoV-2 variants and could support continuous genomic surveillance of SARS-CoV-2 in Indonesia.

## Figures and Tables

**Table 1:** Baseline characteristics of the 292 patients who tested positive for SARS-CoV-2.

Characteristic	Observation (*n* = 292)
Age [years, median (IQR)]	32.0 (26.0–45.0)
Age group [*n* (%)]	
Children and teens (<18 years)	14 (4.8%)
Young adults (18–40 years)	179 (61.3%)
Adults (41–65 years)	85 (29.1%)
Elderly (>65 years)	14 (4.8%)
Sex [*n* (%)]	
Female	140 (47.9%)
Male	152 (52.1%)
Hospital location [*n* (%)]	
Bangka Belitung	35 (12.0%)
Central Kalimantan	52 (17.8%)
East Nusa Tenggara	23 (7.9%)
Greater Jakarta (Jabodetabek)	58 (19.9%)
Jambi	38 (13.0%)
Maluku	36 (12.3%)
North Sulawesi	32 (11.0%)
West Java	18 (6.16%)
Sample type [*n* (%)]	
Nasopharyngeal swab	292 (100%)
Collection month [*n* (%)]	
January	43 (14.7%)
February	45 (15.4%)
March	9 (3.1%)
April	1 (0.3%)
May	4 (1.4%)
June	53 (18.2%)
July	120 (41.1%)
August	16 (5.5%)
October	1 (0.3%)
Hospitalized [*n* (%)]	
Yes	56 (19.2%)
Unknown	236 (80.8%)
Other conditions [*n* (%)]	
Breakthrough infection ^1^	32 (11.0%)
Prolonged viral shedding ^2^	5 (1.7%)
Reinfection	1 (0.3%)
Breakthrough infection and prolonged viral shedding	3 (1.0%)
Breakthrough infection and reinfection	3 (1.0%)
PCR cycle threshold (Ct) value [median (IQR)]	
N gene	18.3 (15.4–21.9)
ORF1ab gene	16.7 (14.7–20.4)

^1^ Patients confirmed positive more than 14 days after the second vaccination dose. ^2^ Viral particles were still detected for over a month after laboratory confirmation.

## Data Availability

The study analyzed 276 whole-genome sequences and metadata available on GISAID at doi.org/10.55876/gis8.230328gx (accessed on 28 March 2023), with the other 16 sequences and metadata available upon request to the corresponding author. We also analyzed 7515 published sequences on GISAID collected between 1 January 2021 and 30 June 2022, available at doi.org/10.55876/gis8.231201wt (accessed on 1 December 2023).

## Supplementary Materials

The following supporting information can be downloaded at https://www.mdpi.com/article/10.3390/pathogens13040279/s1, Figure S1: Characteristics of breakthrough infection, prolonged viral shedding, and reinfection in patients positive for SARS-CoV-2; Figure S2: The distribution of GISAID and Nextstrain clades of SARS-CoV-2 genome from selected Indonesia dataset, comprising 7515 published sequences on GISAID collected from January 1 2021, to June 30 2022; Figure S3: The distribution of mutation events in the spike (S) protein sequence, stratified by SARS-CoV-2 clades; Figure S4: The distribution of mutation events in the nucleocapsid (N) protein sequence, stratified by SARS-CoV-2 clades; Figure S5: Timeline of amino acid substitution in SARS-CoV-2 genomes from Indonesian isolates; Table S1: Baseline characteristics of the studied participant, stratified by region; Table S2: Characteristics of study subjects with breakthrough infection, prolonged viral shedding, and reinfection; Supplemental Table GISAID Identifier: EPI_SET_230328gx; Supplemental Table GISAID Identifier: EPI_SET_231201wt.

## References

[1] WHO Coronavirus (COVID-19) Dashboard. https://covid19.who.int.

[2] Duerr R., Dimartino D., Marier C., Zappile P., Levine S., Francois F., Iturrate E., Wang G., Dittmann M., Lighter J. (2022). Clinical and Genomic Signatures of SARS-CoV-2 Delta Breakthrough Infections in New York. eBioMedicine.

[3] Shahapur P.R., Shahapur R., Bagali S., Karigoudar R., Wavare D.S.P.J., Kandi V., Suvvari T.K., Mittal R.J., Jadhav M. (2022). Breakthrough Infections: Clinical Profile and Outcomes of COVID-19 Vaccinated and Unvaccinated People from a Tertiary Care Hospital. Cureus.

[4] Puhach O., Meyer B., Eckerle I. (2023). SARS-CoV-2 Viral Load and Shedding Kinetics. Nat. Rev. Microbiol..

[5] Naqvi A.A.T., Fatima K., Mohammad T., Fatima U., Singh I.K., Singh A., Atif S.M., Hariprasad G., Hasan G.M., Hassan M.I. (2020). Insights into SARS-CoV-2 Genome, Structure, Evolution, Pathogenesis and Therapies: Structural Genomics Approach. Biochim. Biophys. Acta Mol. Basis Dis..

[6] Carabelli A.M., Peacock T.P., Thorne L.G., Harvey W.T., Hughes J., de Silva T.I., Peacock S.J., Barclay W.S., de Silva T.I., Towers G.J. (2023). SARS-CoV-2 Variant Biology: Immune Escape, Transmission and Fitness. Nat. Rev. Microbiol..

[7] Li Q., Wu J., Nie J., Zhang L., Hao H., Liu S., Zhao C., Zhang Q., Liu H., Nie L. (2020). The Impact of Mutations in SARS-CoV-2 Spike on Viral Infectivity and Antigenicity. Cell.

[8] Tosta S., Moreno K., Schuab G., Fonseca V., Segovia F.M.C., Kashima S., Elias M.C., Sampaio S.C., Ciccozzi M., Alcantara L.C.J. (2023). Global SARS-CoV-2 Genomic Surveillance: What We Have Learned (so Far). Infect. Genet. Evol..

[9] Zhu M., Zeng Q., Saputro B.I.L., Chew S.P., Chew I., Frendy H., Tan J.W., Li L. (2022). Tracking the Molecular Evolution and Transmission Patterns of SARS-CoV-2 Lineage B.1.466.2 in Indonesia Based on Genomic Surveillance Data. Virol. J..

[10] Cahyani I., Putro E.W., Ridwanuloh A.M., Wibowo S., Hariyatun H., Syahputra G., Akbariani G., Utomo A.R., Ilyas M., Loose M. (2022). Genome Profiling of SARS-CoV-2 in Indonesia, ASEAN and the Neighbouring East Asian Countries: Features, Challenges and Achievements. Viruses.

[11] Massi M.N., Abidin R.S., Farouk A.-E., Halik H., Soraya G.V., Hidayah N., Sjahril R., Handayani I., Hakim M.S., Gazali F.M. (2022). Full-Genome Sequencing and Mutation Analysis of SARS-CoV-2 Isolated from Makassar, South Sulawesi, Indonesia. PeerJ.

[12] Fibriani A., Stephanie R., Alfiantie A.A., Siregar A.L.F., Pradani G.A.P., Yamahoki N., Purba W.S., Alamanda C.N.C., Rahmawati E., Rachman R.W. (2021). Analysis of SARS-CoV-2 Genomes from West Java, Indonesia. Viruses.

[13] Pradipta A., Kumaheri M.A., Wahyudi L.D., Susanto A.P., Agasi H.I., Shankar A.H., Sudarmono P. (2022). Accelerating Detection of Variants During COVID-19 Surges by Diverse Technological and Public Health Partnerships: A Case Study from Indonesia. Front. Genet..

[14] Bhoyar R.C., Jain A., Sehgal P., Divakar M.K., Sharma D., Imran M., Jolly B., Ranjan G., Rophina M., Sharma S. (2021). High Throughput Detection and Genetic Epidemiology of SARS-CoV-2 Using COVIDSeq next-Generation Sequencing. PLoS ONE.

[15] Trimarsanto H. Ncov19-Pipeline. https://github.com/trmznt/ncov19-pipeline.

[16] Bushnell B. BBTools. https://sourceforge.net/projects/bbmap/.

[17] Martin M. (2011). Cutadapt Removes Adapter Sequences from High-Throughput Sequencing Reads. EMBnet. J..

[18] Li H. (2018). Minimap2: Pairwise Alignment for Nucleotide Sequences. Bioinformatics.

[19] Grubaugh N.D., Gangavarapu K., Quick J., Matteson N.L., De Jesus J.G., Main B.J., Tan A.L., Paul L.M., Brackney D.E., Grewal S. (2019). An Amplicon-Based Sequencing Framework for Accurately Measuring Intrahost Virus Diversity Using PrimalSeq and iVar. Genome Biol..

[20] Khare S., Gurry C., Freitas L., Schultz M.B., Bach G., Diallo A., Akite N., Ho J., Lee R.T., Yeo W. (2021). GISAID’s Role in Pandemic Response. CCDCW.

[21] Rambaut A., Holmes E.C., O’Toole Á., Hill V., McCrone J.T., Ruis C., du Plessis L., Pybus O.G. (2020). A Dynamic Nomenclature Proposal for SARS-CoV-2 Lineages to Assist Genomic Epidemiology. Nat. Microbiol..

[22] GISAID Global Initiative on Sharing All Influenza Data (GISAID) (2021). Clade and Lineage Nomenclature Aids in Genomic Epidemiology Studies of Active hCoV-19 Viruses.

[23] Nextstrain: Genomic Epidemiology of Novel Coronavirus—Global Subsampling. https://nextstrain.org/ncov.

[24] Hadfield J., Megill C., Bell S.M., Huddleston J., Potter B., Callender C., Sagulenko P., Bedford T., Neher R.A. (2018). Nextstrain: Real-Time Tracking of Pathogen Evolution. Bioinformatics.

[25] Mercatelli D., Triboli L., Fornasari E., Ray F., Giorgi F.M. (2021). Coronapp: A Web Application to Annotate and Monitor SARS-CoV-2 Mutations. J. Med. Virol..

[26] Hoan N.X., Pallerla S.R., Huy P.X., Krämer H., My T.N., Tung T.T., Hoan P.Q., Toan N.L., Song L.H., Velavan T.P. (2022). SARS-CoV-2 Viral Dynamics of the First 1000 Sequences from Vietnam and Neighbouring ASEAN Countries. IJID Reg..

[27] Fan Y., Li X., Zhang L., Wan S., Zhang L., Zhou F. (2022). SARS-CoV-2 Omicron Variant: Recent Progress and Future Perspectives. Signal Transduct. Target. Ther..

[28] Zhao Z., Zhou J., Tian M., Huang M., Liu S., Xie Y., Han P., Bai C., Han P., Zheng A. (2022). Omicron SARS-CoV-2 Mutations Stabilize Spike up-RBD Conformation and Lead to a Non-RBM-Binding Monoclonal Antibody Escape. Nat. Commun..

[29] Kumar S., Delipan R., Chakraborty D., Kanjo K., Singh R., Singh N., Siddiqui S., Tyagi A., Jha V., Thakur K.G. (2023). Mutations in S2 Subunit of SARS-CoV-2 Omicron Spike Strongly Influence Its Conformation, Fusogenicity, and Neutralization Sensitivity. J. Virol..

[30] Cherian S., Potdar V., Jadhav S., Yadav P., Gupta N., Das M., Rakshit P., Singh S., Abraham P., Panda S. (2021). SARS-CoV-2 Spike Mutations, L452R, T478K, E484Q and P681R, in the Second Wave of COVID-19 in Maharashtra, India. Microorganisms.

[31] Wilhelm A., Toptan T., Pallas C., Wolf T., Goetsch U., Gottschalk R., Vehreschild M.J.G.T., Ciesek S., Widera M. (2021). Antibody-Mediated Neutralization of Authentic SARS-CoV-2 B.1.617 Variants Harboring L452R and T478K/E484Q. Viruses.

[32] Korber B., Fischer W.M., Gnanakaran S., Yoon H., Theiler J., Abfalterer W., Hengartner N., Giorgi E.E., Bhattacharya T., Foley B. (2020). Tracking Changes in SARS-CoV-2 Spike: Evidence That D614G Increases Infectivity of the COVID-19 Virus. Cell.

[33] Yurkovetskiy L., Wang X., Pascal K.E., Tomkins-Tinch C., Nyalile T.P., Wang Y., Baum A., Diehl W.E., Dauphin A., Carbone C. (2020). Structural and Functional Analysis of the D614G SARS-CoV-2 Spike Protein Variant. Cell.

[34] Zhang L., Jackson C.B., Mou H., Ojha A., Peng H., Quinlan B.D., Rangarajan E.S., Pan A., Vanderheiden A., Suthar M.S. (2020). SARS-CoV-2 Spike-Protein D614G Mutation Increases Virion Spike Density and Infectivity. Nat. Commun..

[35] Liu Y., Liu J., Johnson B.A., Xia H., Ku Z., Schindewolf C., Widen S.G., An Z., Weaver S.C., Menachery V.D. (2022). Delta Spike P681R Mutation Enhances SARS-CoV-2 Fitness over Alpha Variant. Cell Rep..

[36] Planas D., Veyer D., Baidaliuk A., Staropoli I., Guivel-Benhassine F., Rajah M.M., Planchais C., Porrot F., Robillard N., Puech J. (2021). Reduced Sensitivity of SARS-CoV-2 Variant Delta to Antibody Neutralization. Nature.

[37] Mishra T., Dalavi R., Joshi G., Kumar A., Pandey P., Shukla S., Mishra R.K., Chande A. (2022). SARS-CoV-2 Spike E156G/Δ157-158 Mutations Contribute to Increased Infectivity and Immune Escape. Life Sci. Alliance.

[38] Ginex T., Marco-Marín C., Wieczór M., Mata C.P., Krieger J., Ruiz-Rodriguez P., López-Redondo M.L., Francés-Gómez C., Melero R., Sánchez-Sorzano C.Ó. (2022). The Structural Role of SARS-CoV-2 Genetic Background in the Emergence and Success of Spike Mutations: The Case of the Spike A222V Mutation. PLoS Pathog..

[39] Hu L., Tang Y., Mei L., Liang M., Huang J., Wang X., Wu L., Jiang J., Li L., Long F. (2023). A New Intracellular Targeting Motif in the Cytoplasmic Tail of the Spike Protein May Act as a Target to Inhibit SARS-CoV-2 Assembly. Antivir. Res..

[40] Thomson E.C., Rosen L.E., Shepherd J.G., Spreafico R., da Silva Filipe A., Wojcechowskyj J.A., Davis C., Piccoli L., Pascall D.J., Dillen J. (2021). Circulating SARS-CoV-2 Spike N439K Variants Maintain Fitness While Evading Antibody-Mediated Immunity. Cell.

[41] Wu W., Cheng Y., Zhou H., Sun C., Zhang S. (2023). The SARS-CoV-2 Nucleocapsid Protein: Its Role in the Viral Life Cycle, Structure and Functions, and Use as a Potential Target in the Development of Vaccines and Diagnostics. Virol. J..

[42] Moody R., Wilson K.L., Boer J.C., Holien J.K., Flanagan K.L., Jaworowski A., Plebanski M. (2021). Predicted B Cell Epitopes Highlight the Potential for COVID-19 to Drive Self-Reactive Immunity. Front. Bioinform..

[43] Liu W., Li H. (2023). COVID-19: Attacks Immune Cells and Interferences With Antigen Presentation Through MHC-Like Decoy System. J. Immunother..

[44] Alsuwairi F.A., Alsaleh A.N., Alsanea M.S., Al-Qahtani A.A., Obeid D., Almaghrabi R.S., Alahideb B.M., AlAbdulkareem M.A., Mutabagani M.S., Althawadi S.I. (2023). Association of SARS-CoV-2 Nucleocapsid Protein Mutations with Patient Demographic and Clinical Characteristics during the Delta and Omicron Waves. Microorganisms.

[45] Wu H., Xing N., Meng K., Fu B., Xue W., Dong P., Tang W., Xiao Y., Liu G., Luo H. (2021). Nucleocapsid Mutations R203K/G204R Increase the Infectivity, Fitness, and Virulence of SARS-CoV-2. Cell Host Microbe.

[46] Haddad D., John S.E., Mohammad A., Hammad M.M., Hebbar P., Channanath A., Nizam R., Al-Qabandi S., Madhoun A.A., Alshukry A. (2021). SARS-CoV-2: Possible Recombination and Emergence of Potentially More Virulent Strains. PLoS ONE.

[47] Chaudhari A., Chaudhari M., Mahera S., Saiyed Z., Nathani N.M., Shukla S., Patel D., Patel C., Joshi M., Joshi C.G. (2021). In-Silico Analysis Reveals Lower Transcription Efficiency of C241T Variant of SARS-CoV-2 with Host Replication Factors MADP1 and hnRNP-1. Inform. Med. Unlocked.

[48] Hosseini R.S.M.A., McLellan A.D. (2020). Implications of SARS-CoV-2 Mutations for Genomic RNA Structure and Host microRNA Targeting. Int. J. Mol. Sci..

[49] Periwal N., Rathod S.B., Sarma S., Johar G.S., Jain A., Barnwal R.P., Srivastava K.R., Kaur B., Arora P., Sood V. (2022). Time Series Analysis of SARS-CoV-2 Genomes and Correlations among Highly Prevalent Mutations. Microbiol. Spectr..

[50] Lopez B.J., Andrews N., Gower C., Gallagher E., Simmons R., Thelwall S., Stowe J., Tessier E., Groves N., Dabrera G. (2021). Effectiveness of Covid-19 Vaccines against the B.1.617.2 (Delta) Variant. N. Engl. J. Med..

[51] Fowlkes A., Gaglani M., Groover K., Thiese M.S., Tyner H., Ellingson K. (2021). Effectiveness of COVID-19 Vaccines in Preventing SARS-CoV-2 Infection Among Frontline Workers Before and During B.1.617.2 (Delta) Variant Predominance—Eight U.S. Locations, December 2020–August 2021. Morb. Mortal. Wkly. Rep..

[52] Mengist H.M., Kombe K.A.J., Mekonnen D., Abebaw A., Getachew M., Jin T. (2021). Mutations of SARS-CoV-2 Spike Protein: Implications on Immune Evasion and Vaccine-Induced Immunity. Semin. Immunol..

[53] Deng X., Garcia-Knight M.A., Khalid M.M., Servellita V., Wang C., Morris M.K., Sotomayor-González A., Glasner D.R., Reyes K.R., Gliwa A.S. (2021). Transmission, Infectivity, and Neutralization of a Spike L452R SARS-CoV-2 Variant. Cell.

[54] Motozono C., Toyoda M., Zahradnik J., Saito A., Nasser H., Tan T.S., Ngare I., Kimura I., Uriu K., Kosugi Y. (2021). SARS-CoV-2 Spike L452R Variant Evades Cellular Immunity and Increases Infectivity. Cell Host Microbe.

[55] Wall E.C., Wu M., Harvey R., Kelly G., Warchal S., Sawyer C., Daniels R., Hobson P., Hatipoglu E., Ngai Y. (2021). Neutralising Antibody Activity against SARS-CoV-2 VOCs B.1.617.2 and B.1.351 by BNT162b2 Vaccination. Lancet.

[56] Niyonkuru M., Pedersen R.M., Assing K., Andersen T.E., Skov M.N., Johansen I.S., Madsen L.W. (2021). Prolonged Viral Shedding of SARS-CoV-2 in Two Immunocompromised Patients, a Case Report. BMC Infect. Dis..

[57] Hossain M.E., Lister D., Bartolo C., Kinsella P.M., Knox J., Aldrich R., Cowan R., Commons R.J. (2021). Prolonged Viral Shedding in Patients with Mild to Moderate COVID-19 Disease: A Regional Perspective. Infect. Dis..

[58] Long H., Zhao J., Zeng H.-L., Lu Q.-B., Fang L.-Q., Wang Q., Wu Q.-M., Liu W. (2021). Prolonged Viral Shedding of SARS-CoV-2 and Related Factors in Symptomatic COVID-19 Patients: A Prospective Study. BMC Infect. Dis..

[59] Shen L., Triche T.J., Bard J.D., Biegel J.A., Judkins A.R., Gai X. (2021). Spike Protein NTD Mutation G142D in SARS-CoV-2 Delta VOC Lineages Is Associated with Frequent Back Mutations, Increased Viral Loads, and Immune Evasion. Medrxiv.

[60] Siedner M.J., Boucau J., Gilbert R.F., Uddin R., Luu J., Haneuse S., Vyas T., Reynolds Z., Iyer S., Chamberlin G.C. (2022). Duration of Viral Shedding and Culture Positivity with Postvaccination SARS-CoV-2 Delta Variant Infections. JCI Insight.

[61] Lee J.-S., Yun K.W., Jeong H., Kim B., Kim M.J., Park J.H., Shin H.S., Oh H.S., Sung H., Song M.G. (2022). SARS-CoV-2 Shedding Dynamics and Transmission in Immunosuppressed Patients. Virulence.

[62] Ling-Hu T., Rios-Guzman E., Lorenzo-Redondo R., Ozer E.A., Hultquist J.F. (2022). Challenges and Opportunities for Global Genomic Surveillance Strategies in the COVID-19 Era. Viruses.

[63] Silk B.J. (2023). COVID-19 Surveillance After Expiration of the Public Health Emergency Declaration—United States, May 11, 2023. MMWR Morb. Mortal. Wkly. Rep..

[64] Jawad B., Adhikari P., Podgornik R., Ching W.-Y. (2023). Impact of BA.1, BA.2, and BA.4/BA.5 Omicron Mutations on Therapeutic Monoclonal Antibodies. Comput. Biol. Med..

