# Supplementary material for: Whole-Genome Sequencing and Mutation Analyses of SARS-CoV-2 Isolates from Indonesia
Source: Pathogens. 2024 Mar 25;13(4):279. doi: 10.3390/pathogens13040279 (PMC11053823; doi:10.3390/pathogens13040279)
Supplement: Supplementary file 1 [file pathogens-13-00279-s001.zip › pathogens-2730849-supplementary.pdf]

pathogens

MDPI

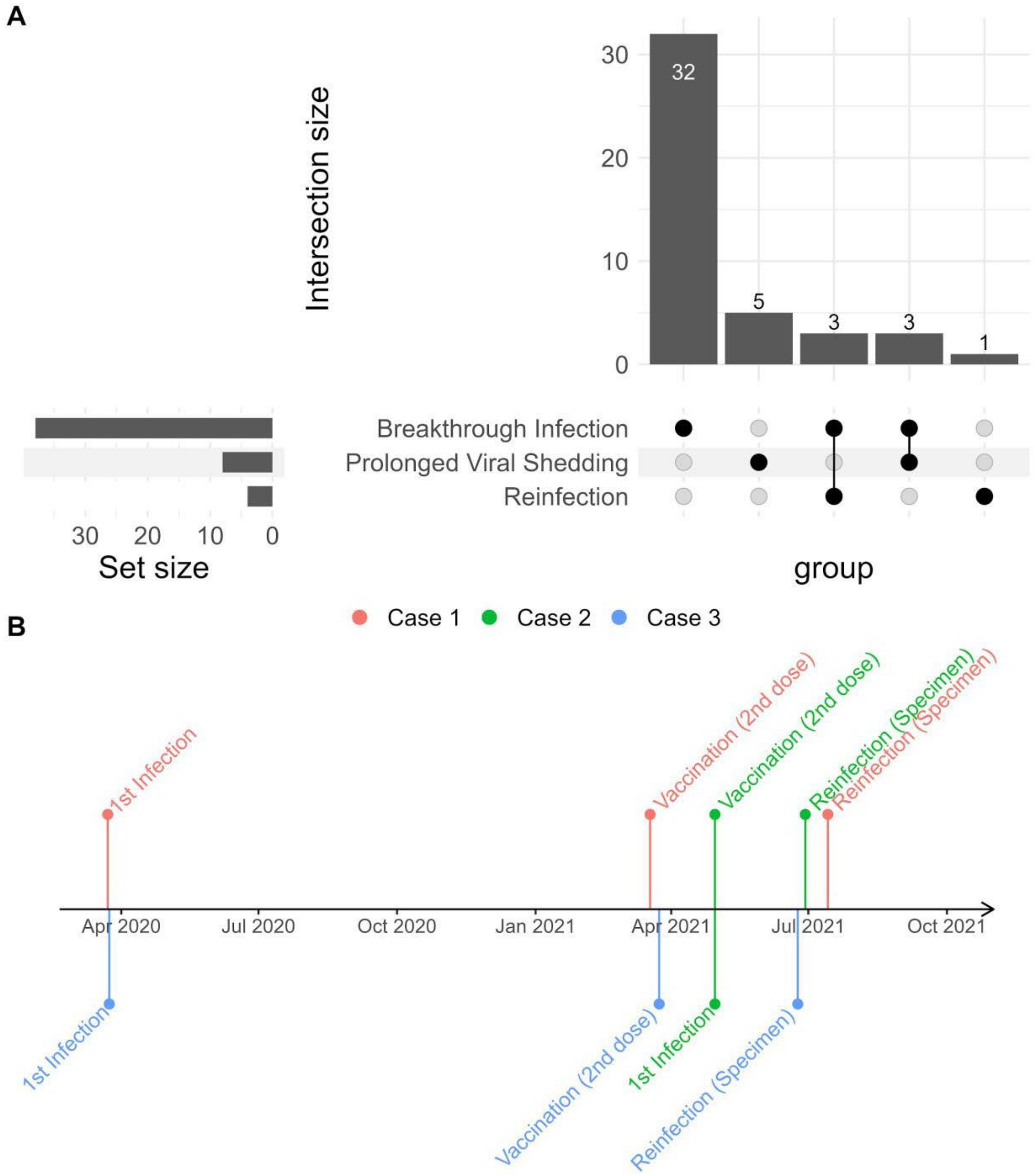

Figure S1. Characteristics of breakthrough infection, prolonged viral shedding, and reinfection in patients positive for SARS-CoV-2. (**A**) An upset plot showing the co-occurrence of these cases. (**B**) Timeline of first and second infections and vaccination dates among three cases with combined breakthrough infection and reinfection.

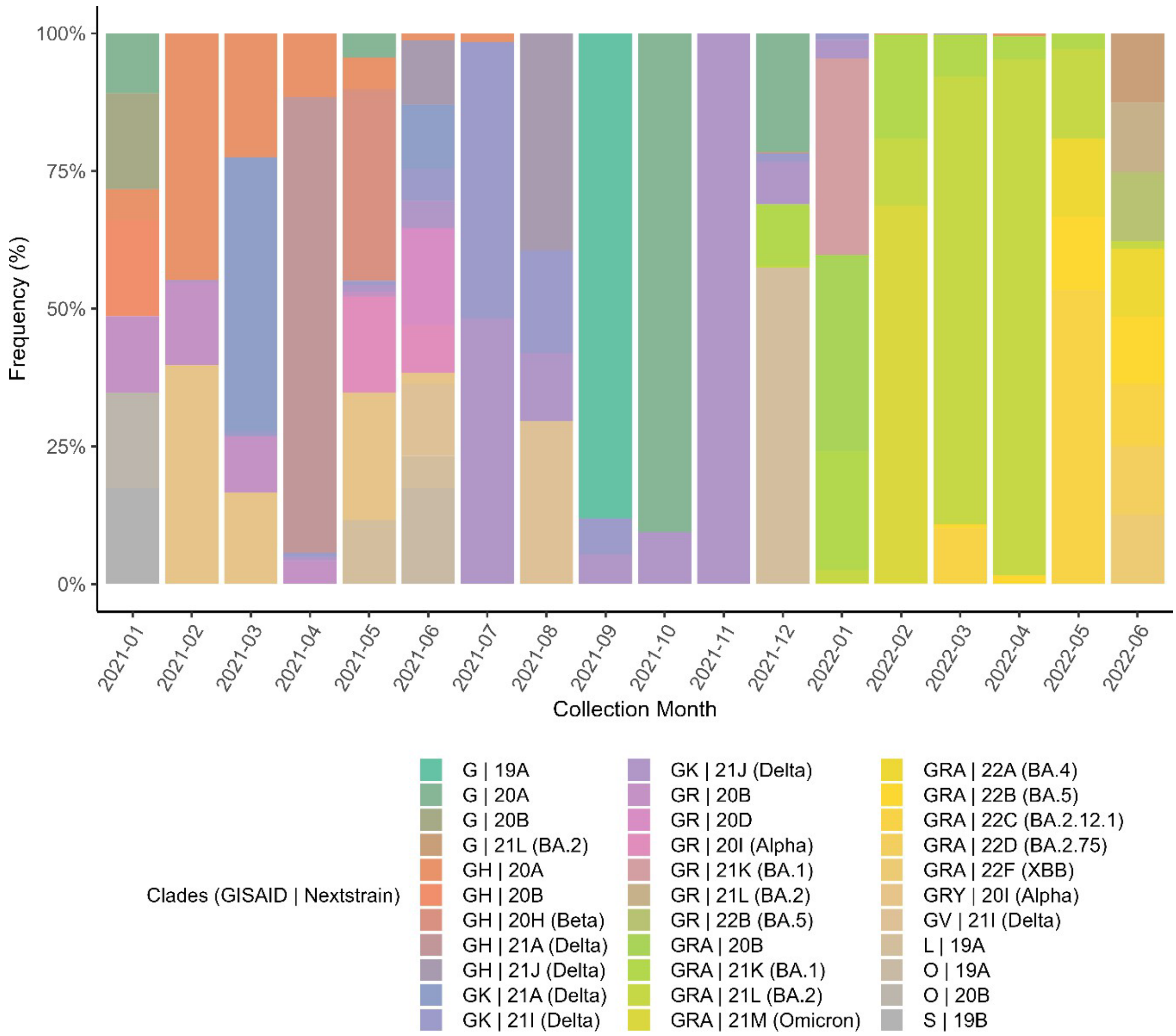

Figure S2. The distribution of GISAID and Nextstrain clades of SARS-CoV-2 genome from selected Indonesia dataset, comprising 7,515 published sequences on GISAID collected from January 1, 2021, to June 30, 2022. These sequences belonged to respective regions similar to the study site, specifically from Bangka Belitung (*n* = 159), Central Kalimantan (*n* = 224), East Nusa Tenggara (*n* = 280), Greater Jakarta/Jabodetabek (*n* = 4,017), Jambi (*n* = 679), Maluku (*n* = 79), North Sulawesi (*n* = 258), and West Java (*n* = 1,819). The associated dataset is available on GISIAD at doi.org/10.55876/ gis8.231201wt (accessed on December 1, 2023).

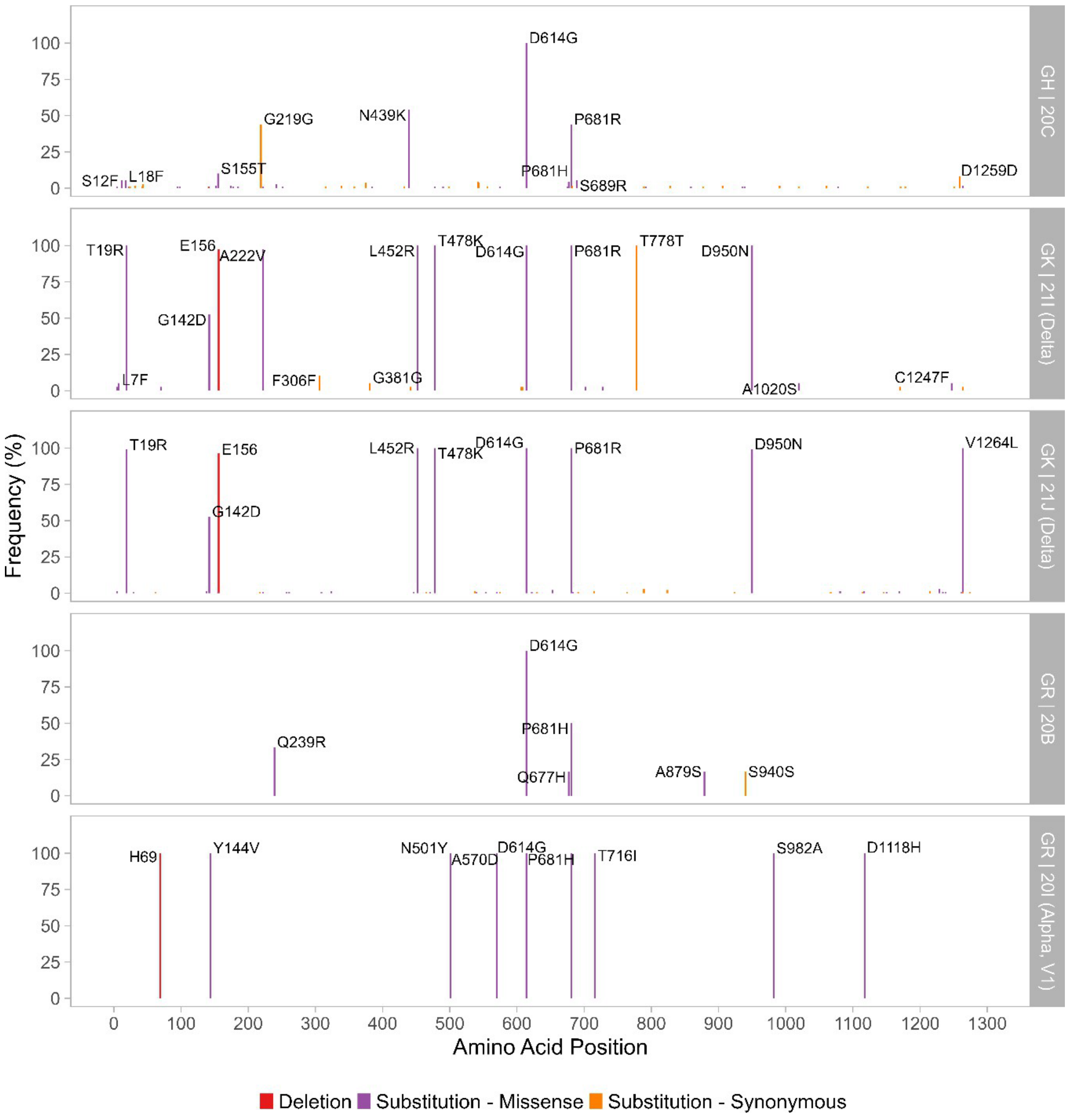

Figure S3. The distribution of mutation events in the spike (S) protein sequence, stratified by SARS-CoV-2 clades. Mutations with a frequency of >5% are indicated.

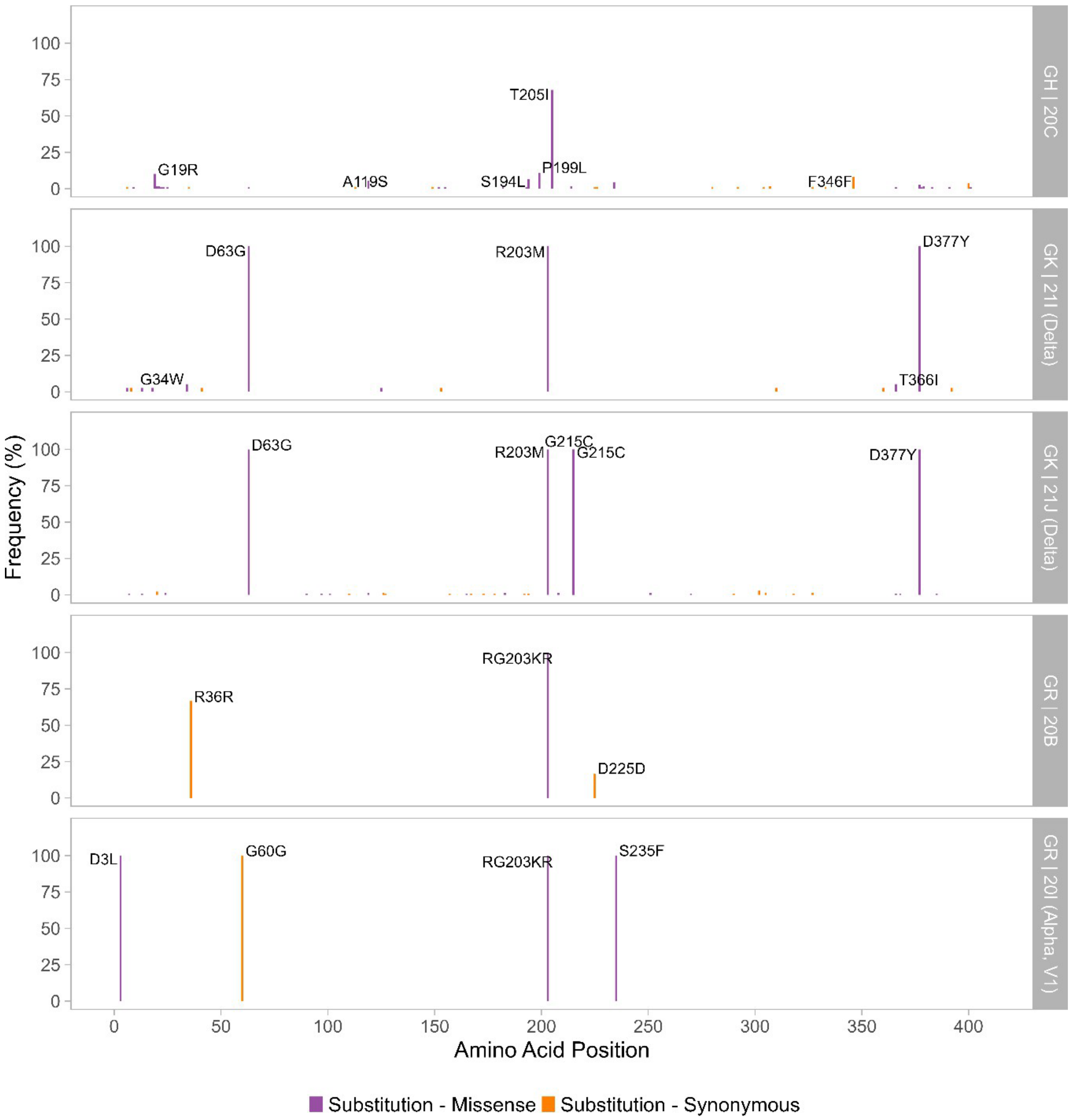

Figure S4. The distribution of mutation events in the nucleocapsid (N) protein sequence, stratified by SARS-CoV-2 clades. Mutations with a frequency of >5% are indicated.

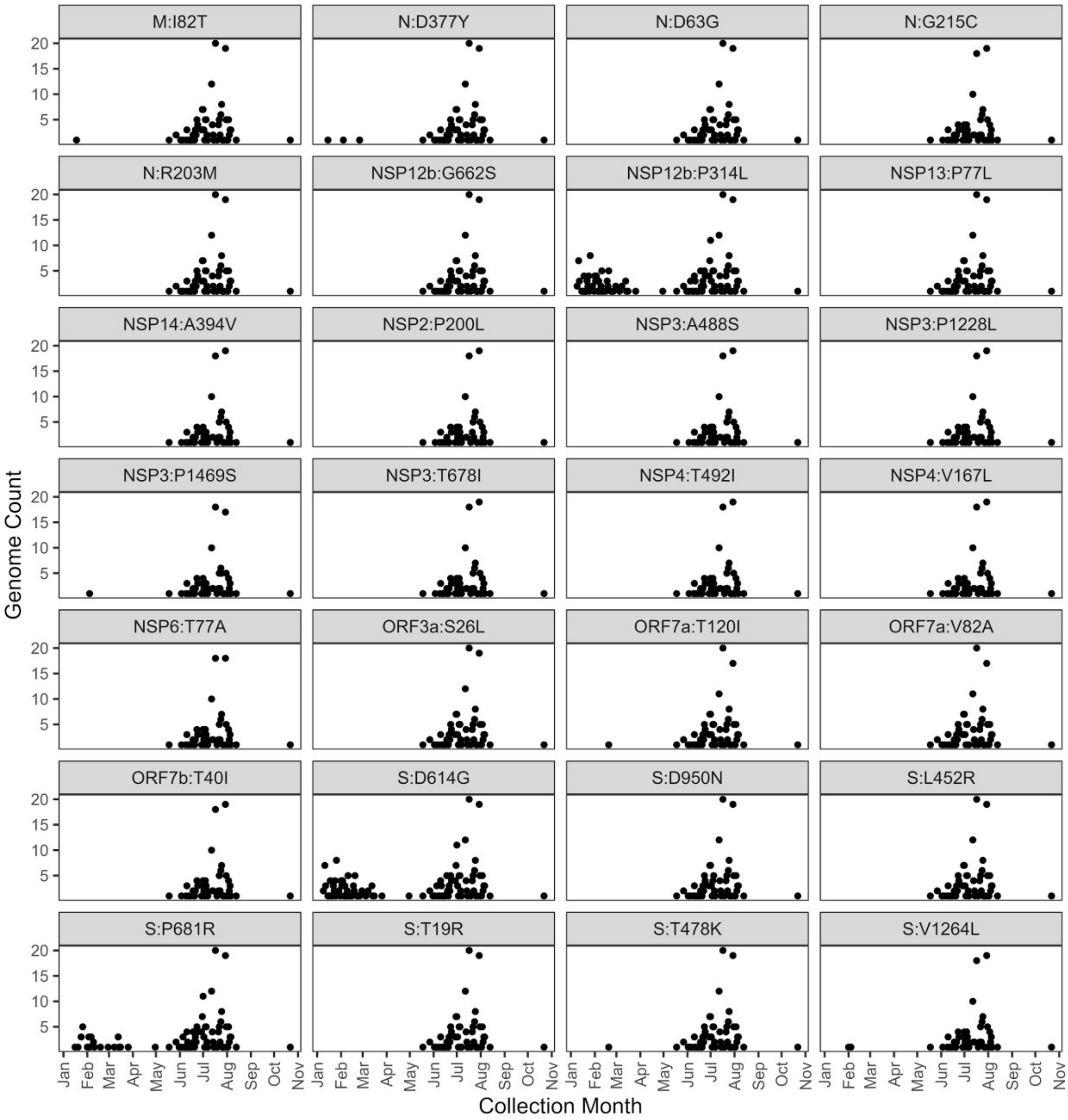

Figure S5. Timeline of amino acid substitution in SARS-CoV-2 genomes from Indonesian isolates.

Table S1. Baseline characteristics of the studied participant, stratified by region.

**Characteristic**		**Bangka Belitung (*n* = 35)**	**Central Kalimantan (*n* = 52)**	**East Nusa Tenggara (*n* = 23)**	**Greater Jakarta (*n* = 58)**	**Jambi (*n* = 38)**	**Maluku (*n* = 36)**	**North Sulawesi (*n* = 32)**	**West Java (*n* = 18)**
Age	Median (IQR)	41 (28-50)	33.5 (28-45.2)	30 (24-32.5)	30 (24.2-37.8)	31 (27-47)	34.5 (25.5-46)	38.5 (30-53.2)	34 (28-47.8)
Sex	Female	18 (51%)	24 (46%)	8 (35%)	33 (57%)	18 (47%)	14 (39%)	17 (53%)	8 (44%)
	Male	17 (49%)	28 (54%)	15 (65%)	25 (43%)	20 (53%)	22 (61%)	15 (47%)	10 (56%)
Collection month	January	2 (6%)	6 (12%)	14 (61%)	3 (5%)	5 (13%)	0 (0%)	7 (22%)	6 (33%)
	February	4 (11%)	14 (27%)	9 (39%)	1 (2%)	4 (11%)	0 (0%)	1 (3%)	12 (67%)
	March	0 (0%)	1 (2%)	0 (0%)	8 (14%)	0 (0%)	0 (0%)	0 (0%)	0 (0%)
	April	0 (0%)	0 (0%)	0 (0%)	1 (2%)	0 (0%)	0 (0%)	0 (0%)	0 (0%)
	May	1 (3%)	0 (0%)	0 (0%)	3 (5%)	0 (0%)	0 (0%)	0 (0%)	0 (0%)
	June	4 (11%)	21 (40%)	0 (0%)	24 (41%)	4 (11%)	0 (0%)	0 (0%)	0 (0%)
	July	22 (63%)	10 (19%)	0 (0%)	15 (26%)	25 (66%)	36 (100%)	12 (38%)	0 (0%)
	August	2 (6%)	0 (0%)	0 (0%)	2 (3%)	0 (0%)	0 (0%)	12 (38%)	0 (0%)
	October	0 (0%)	0 (0%)	0 (0%)	1 (2%)	0 (0%)	0 (0%)	0 (0%)	0 (0%)
Hospitalized	Yes	6 (17%)	16 (31%)	0 (0%)	5 (9%)	2 (5%)	0 (100%)	25 (78%)	2 (11%)
	Unknown	29 (83%)	36 (69%)	23 (100%)	53 (91%)	36 (95%)	36 (0%)	7 (22%)	16 (89%)
Other conditions	BI [1]	0 (0%)	0 (0%)	0 (0%)	32 (55%)	0 (0%)	0 (0%)	0 (0%)	0 (0%)
	PVS [2]	0 (0%)	0 (0%)	0 (0%)	5 (9%)	0 (0%)	0 (0%)	0 (0%)	0 (0%)
	RI [3]	0 (0%)	0 (0%)	0 (0%)	1 (2%)	0 (0%)	0 (0%)	0 (0%)	0 (0%)
	BI+PVS	0 (0%)	0 (0%)	0 (0%)	3 (5%)	0 (0%)	0 (0%)	0 (0%)	0 (0%)
	BI+RI	0 (0%)	0 (0%)	0 (0%)	3 (5%)	0 (0%)	0 (0%)	0 (0%)	0 (0%)

1 BI, breakthrough infection. Patients confirmed positive more than 14 days after the second dose of vaccination.

2 PVS, prolonged viral shedding. Viral particles were still detected for over a month after laboratory confirmation.

3 RI, reinfection.

Table S2. Characteristics of study subjects with breakthrough infection, prolonged viral shedding, and reinfection.

**Characteristic**		**BI [1] (*n* = 32)**	**PVS [2] (*n* = 5)**	**RI [3] (*n* = 1)**	**BI+PVS (*n* = 3)**	**BI+RI (*n* = 3)**
Age	Median (IQR)	26 (24-29)	41 (39-45)	41	28 (27-29.5)	41 (33.5-45)
Sex	Female	21 (66%)	1 (20%)	1 (100%)	2 (67%)	3 (100%)
	Male	11 (34%)	4 (80%)	0 (0%)	1 (33%)	0 (0%)
Hospital location	Greater Jakarta	32 (100%)	5 (100%)	1 (100%)	3 (100%)	3 (100%)
Collection month	March	2 (6%)	0 (0%)	0 (0%)	0 (0%)	0 (0%)
	April	0 (0%)	0 (0%)	1 (100%)	0 (0%)	0 (0%)
	June	17 (53%)	4 (80%)	0 (0%)	1 (33%)	2 (67%)
	July	12 (38%)	1 (20%)	0 (0%)	1 (33%)	1 (33%)
	August	1 (3%)	0 (0%)	0 (0%)	1 (33%)	0 (0%)
Hospitalized	Yes	1 (3%)	0 (0%)	0 (0%)	1 (33%)	1 (33%)
	Unknown	31 (97%)	5 (100%)	1 (100%)	2 (67%)	2 (67%)
Fully vaccinated	Yes	32 (100%)	5 (100%)	0 (0%)	3 (100%)	3 (100%)
	No	0 (%)	0 (%)	0 (0%)	0 (0%)	0 (0%)
	NA	0 (%)	0 (%)	1 (100%)	0 (0%)	0 (0%)
BI intervals (days)	Median (IQR)	127.5 (111.5-139.5)	NA	NA	NA	92 (76-105)
Median PCR Ct value	N gene	18.0 (16.2-21.3)	14.7 (14.4-18.3)	15.3	16.4 (15.2-20.1)	18.1 (17.7-23.9)
	ORF1ab gene	17.2 (14.9-20.2)	14.7 (13.0-18.2)	16.8	15.6 (14.4-18.5)	17.5 (17.3-22.7)
Clade	GH ǀ 20C	2 (6.3%)	0 (%)	1 (100%)	0 (0%)	0 (0%)
	GK ǀ 21J (Delta)	18 (56.2%)	5 (100%)	0 (0%)	3 (100%)	2 (67%)
	GK ǀ 21I (Delta)	12 (37.5%)	0 (%)	0 (0%)	0 (0%)	1 (33%)
Lineage	B.1.466.2	2 (6.2%)	0 (%)	1 (100%)	0 (0%)	0 (0%)
	AY.23	18 (56.3%)	5 (100%)	0 (0%)	3 (100%)	2 (67%)
	AY.24	12 (37.5%)	0 (%)	0 (0%)	0 (0%)	1 (33%)

[1] BI, breakthrough infection. Patients confirmed positive more than 14 days after the second dose of vaccination.

[2] PVS, prolonged viral shedding. Viral particles were still detected for over a month after laboratory confirmation.

[3] RI, reinfection.

NA: data not available.

SUPPLEMENTAL TABLE

Data Availability

GISAID Identifier: EPI_SET_230328gx
doi: 10.55876/gis8.230328gx

All genome sequences and associated metadata in this dataset are published in GISAID's EpiCoV database. To view the contributors of each individual sequence with details such as accession number, Virus name, Collection date, Originating Lab and Submitting Lab and the list of Authors, visit 10.55876/gis8.230328gx

Data Snapshot

- EPI_SET_230328gx is composed of 276 individual genome sequences.
- The collection dates range from 2021-01-09 to 2021-10-22;
- Data were collected in 1 countries and territories;
- All sequences in this dataset are compared relative to hCoV-19/Wuhan/WIV04/2019 (WIV04), the official reference sequence employed by GISAID (EPI_ISL_402124). Learn more at https://gisaid.org/WIV04.

SUPPLEMENTAL TABLE

Data Availability

GISAID Identifier:	EPI_SET_231201wt
doi:	10.55876/gis8.231201wt

All genome sequences and associated metadata in this dataset are published in GISAID's EpiCoV database. To view the contributors of each individual sequence with details such as accession number, Virus name, Collection date, Originating Lab and Submitting Lab and the list of Authors, visit 10.55876/gis8.231201wt

Data Snapshot

- EPI_SET_231201wt is composed of 7,515 individual genome sequences.
- The collection dates range from 2021-01-01 to 2022-06-30;
- Data were collected in 1 countries and territories;
- All sequences in this dataset are compared relative to hCoV-19/Wuhan/WIV04/2019 (WIV04), the official reference sequence employed by GISAID (EPI_ISL_402124). Learn more at https://gisaid.org/WIV04.
